# Toolbox for Genetic Transformation of Non-Conventional *Saccharomycotina* Yeasts: High Efficiency Transformation of Yeasts Belonging to the *Schwanniomyces* Genus

**DOI:** 10.3390/jof8050531

**Published:** 2022-05-20

**Authors:** Angela Matanović, Kristian Arambašić, Bojan Žunar, Anamarija Štafa, Marina Svetec Miklenić, Božidar Šantek, Ivan-Krešimir Svetec

**Affiliations:** Department of Biochemical Engineering, Faculty of Food Technology and Biotechnology, Pierottijeva 6, 10000 Zagreb, Croatia; amatanovic@pbf.hr (A.M.); kristian.arambasic@outlook.com (K.A.); bzunar@pbf.hr (B.Ž.); anamarija.stafa@gmail.com (A.Š.); mmiklenic@pbf.hr (M.S.M.); bsantek@pbf.hr (B.Š.)

**Keywords:** non-conventional yeasts, *Saccharomycotina* subphylum, *Schwanniomyces* species, genetic transformation, electroporation, yeast plasmids

## Abstract

Non-conventional yeasts are increasingly being investigated and used as producers in biotechnological processes which often offer advantages in comparison to traditional and well-established systems. Most biotechnologically interesting non-conventional yeasts belong to the *Saccharomycotina* subphylum, including those already in use (*Pichia pastoris, Yarrowia lypolitica*, etc.), as well as those that are promising but as yet insufficiently characterized. Moreover, for many of these yeasts the basic tools of genetic engineering needed for strain construction, including a procedure for efficient genetic transformation, heterologous protein expression and precise genetic modification, are lacking. The first aim of this study was to construct a set of integrative and replicative plasmids which can be used in various yeasts across the *Saccharomycotina* subphylum. Additionally, we demonstrate here that the electroporation procedure we developed earlier for transformation of *B. bruxellensis* can be applied in various yeasts which, together with the constructed plasmids, makes a solid starting point when approaching a transformation of yeasts form the *Saccharomycotina* subphylum. To provide a proof of principle, we successfully transformed three species from the *Schwanniomyces* genus (*S. polymorphus var. polymorphus*, *S. polymorphus var. africanus* and *S. pseudopolymorphus*) with high efficiencies (up to 8 × 10^3^ in case of illegitimate integration of non-homologous linear DNA and up to 4.7 × 10^5^ in case of replicative plasmid). For the latter two species this is the first reported genetic transformation. Moreover, we found that a plasmid carrying replication origin from *Scheffersomyces stipitis* can be used as a replicative plasmid for these three *Schwanniomyces* species.

## 1. Introduction

In recent years non-conventional yeasts, i.e., all those yeast species beyond well-established model organisms *Saccharomyces cerevisiae* and *Schizosaccharomyces pombe,* are rapidly gaining the interest of researchers and industry [1]. It is becoming clear that the diversity of yeasts offers vast resources in terms of new compounds and biochemical pathways, as well as other biotechnologically interesting features such as thermotolerance, halotolerance, etc. For example, *Yarrowia lipolytica* has been used in single-cell protein production from hydrocarbons and in production of citric acid and lipases also produced by *Candida cylndracea*, *Eremothecium gossypii* is a producer of riboflavin, *Kluyveromyces marxianus* produces lactase, *Komatogella (Pichia) pastoris* and *Ogatea (Hansenula) polymorpha* are important systems for heterologous protein expression, while *Pachysolen tannophilus, Scheffersomyces (Pichia) stipitis, Candida shehatae, Spathaspora passalidarum* and *Candida jeffriesii* can ferment D-xylose to ethanol and are being researched as possible producers of bioethanol from plant biomass [2]. Most of the biotechnologically interesting yeast species, both those that are intensely researched and already in use and those yet poorly investigated but promising, belong to the *Saccharomycotina* subphylum of ascomycetous yeasts [2].

One of the main issues in the fundamental research and biotechnological application of non-convectional yeasts is that the basic tools for genetic transformation and modification are often lacking. Ideally, to utilize the full potential of various yeast species it is necessary to transform them genetically with reasonable efficiency and select transformants, to introduce and express heterologous genes harbored on a replicative plasmid and, ultimately, to precisely modify or at least inactivate desired genes in their genome. The first aim of this work was to establish a set of plasmids with appropriate antibiotic selectable markers and potentially functional replication origins coupled with a transformation protocol which might be fairly broadly applicable in various species belonging to the *Saccharomycotina* subphylum. Although, understandably, it is impossible to predict if a certain replicative plasmid or a certain transformation procedure will be appropriate for a specific species, it is our goal to provide tools which can serve as the best possible starting point when dealing with genetic transformation of a new and unexplored species from the *Saccharomycotina* subphylum. Certainly, in individual cases, further optimizations and modifications might be necessary. We combined four antibiotic selectable markers which were codon-optimized to be functional in species across the *Saccaroymcotina* subphylum (both in species using standard genetic code and those with the CTG clade in which CTG encodes serine instead of leucine), with five replication origins cloned from five species belonging to this subphylum (*Saccharomyces cerevisiae*, *Kluyveromyces lactis, Scheffersomyces stipitis*, *Merozyma guilliermondi* and *Brettanomyces bruxellensis*). Additionally, we demonstrated that the electroporation procedure which we developed in our earlier work [3] for transformation of *B. bruxellensis* can be used for transformation of all five of these different yeast species.

Furthermore, to test the principle that the constructed set of plasmids coupled with the previously developed electroporation protocol will provide a good basis when approaching transformation of other yeasts within the *Saccharomycotina* subphylum, we attempted transformation of three selected species from the *Schwanniomyces* genus—*S. pseudopolymorphus, S. polymorphus var. polymorphus* and *S. polymorphus var. africanus*. This is a fairly poorly explored genus of budding yeasts within the *Saccharomycotina* subphylum, but could have interesting biotechnological potential. The most prominent member of this genus, *S. occidentalis var. occidentalis,* has interesting properties which make it especially suitable for heterologous gene expression [4]. Furthermore, most members of this genus can grow on D-xylose and celobiose [5], making them potentially interesting in the processing of lignocellulose materials for production of various biochemicals. Although genetic transformation of *S. occidentalis var. occidentalis* and *S. polymorphus var. polymorphus* has been reported [6,7,8,9], research on yeasts belonging to the *Schwanniomyces* genus has progressed relatively slowly, possibly since it went unrecognized that this genus is likely to lie within the CTG clade and requires codon optimization of heterologous genes [10]. In this work we managed to transform all three selected *Schwanniomyces* species using the electroporation procedure we described earlier [3] and the plasmid set constructed in this work. For *S. pseudopolymorphus* and *S. polymorphus var. africanus*, this is the first reported genetic transformation. For all three species high transformation efficiencies were achieved. Additionally, we found that plasmid carrying replication origin from *S. stipitis* can be used as a replicative vector in three selected *Schwanniomyces* species, especially in *S. polymorphus var. africanus,* where the stability of this plasmid is 100%. Taken together, we believe the tools and results described in this study will facilitate research and biotechnological application of yeasts belonging to the *Schwanniomycces* genus, as well as other non-conventional budding yeasts from the *Saccharomycotina* subphylum.

## 2. Materials and Methods

### 2.1. Growth Conditions

*Escherichia coli* was grown at 37 °C and 150 rpm in LB-Miller medium (10 g L^−1^ of bacto tryptone, 5 g L^−1^ of yeast extract, 10 g L^−1^ of NaCl), with solid medium also containing 15 g L^−1^ of agar. Transformants were selected for, and plasmids maintained with ampicillin (50 µg mL^−1^ in broth, 100 µg mL^−1^ on solid media; Fisher BioReagents, Pittsburgh, PA, USA).

Yeasts were grown at 28 °C and 150 rpm in YPD medium (10 g L^−1^ of yeast extract, 20 g L^−1^ of bacto peptone, 20 g L^−1^ of glucose), with solid medium also containing 20 g L^−1^ of agar. The transformants were selected with antibiotic concentrations shown in Table 1.

### 2.2. Yeast Strains, Plasmids and Oligonucleotides

Yeast strains, plasmids and oligonucleotides used in this study are listed in Table 2.

### 2.3. Gene Optimisation, Synthesis and Testing

Gene sequences were generated with Integrated DNA Technologies’ (IDT) Codon Optimization Tool, with their open reading frames first optimized to match codon usage of Candida albicans, and then edited further, as described in the Results. Genes were synthesized by GENEWIZ (South Plainfield, NJ, USA). The functionality of de novo synthesized antibiotic resistance markers was verified in *E. coli* at 37 °C on solid LB-Luria plates (10 g L^−1^ of bacto tryptone, 5 g L^−1^ of yeast extract, 0.5 g L^−1^ of NaCl, 15 g L^−1^ of agar) supplemented with 100 µg mL^−1^ of ampicillin and one of the following antibiotics: 50 µg mL^−1^ of kanamycin (BioBasic, Toronto, ON, Canada), 150 µg mL^−1^ of hygromycin B (Fisher Scientific International LLC, Waltham, MA, USA), 50 µg mL^−1^ of clonNAT (Jena Bioscience, Jena, Thuringia, Germany), or 20 µg mL^−1^ of phleomycin (Fisher Scientific International LLC, Waltham, MA, USA).

### 2.4. Plasmid Construction

#### 2.4.1. Construction of Integrative Plasmids

**Figure 1:**
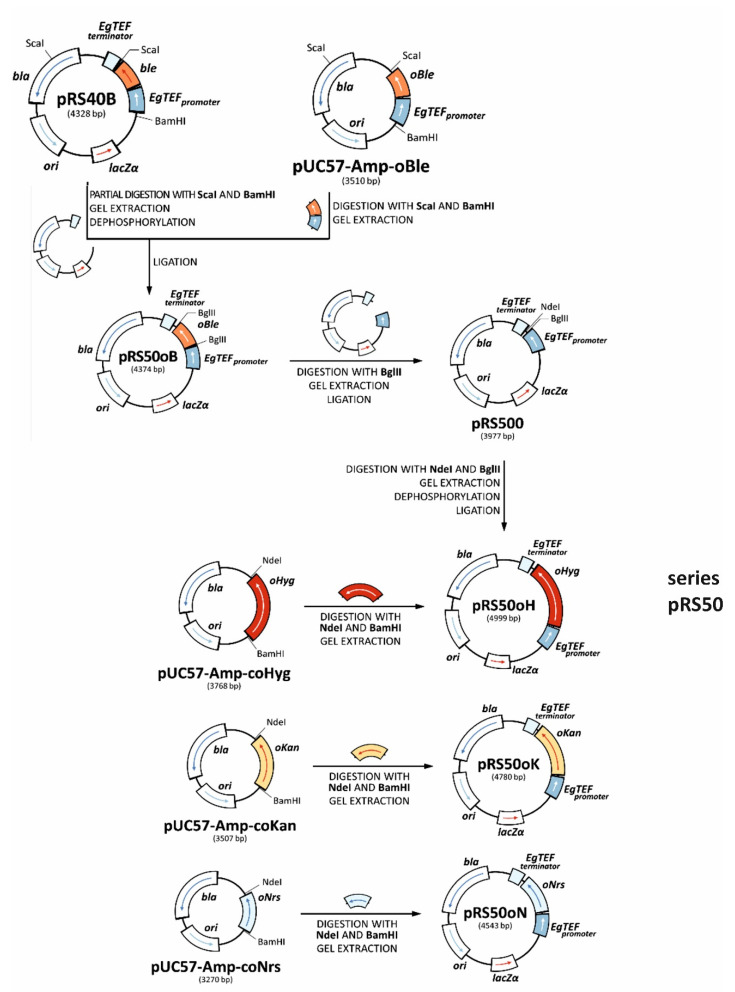
Construction of the plasmid series pRS50 (integrative plasmids) with a detailed list of intermediate cloning steps and relevant restriction sites. ble = original ble ORF from Klebsiella pneumoniae, EgTEFpromoter = promoter of the Eremothecium gossypii TEF gene, EgTEFterminator = terminator of the E. gossypii TEF gene, oBle = codon-optimized ble ORF encoding for resistance to phleomycin, oHyg = codon-optimized ORF encoding for resistance to hygromycin B, oKan = codon-optimized ORF encoding for resistance to G418, oNrs = codon-optimized ORF encoding for resistance to clonNAT, bla = resistance to ampicillin, ori = replication origin from the *E. coli* plasmid pBR322, lacZα = gene encoding α-peptide required for the blue-white screening.

Plasmids in this work were constructed using pRS40B as a starting point [13]. The backbone of pRS40B (Figure 1) carries sequences relevant for transformation and maintenance in *E. coli* (*bla, ori*) and other common features (*LacZα, MCS, f1 ori*), as well as a TEF promoter and terminator originating from *Eremothecium gossypii,* known to be able to drive gene expression in various yeast species [14]. Under the control of the TEF promoter, dominant antibiotic resistance markers needed for selection of yeast transformants and plasmid maintenance in yeast cells were cloned. Markers were codon-optimized to be functional across the *Saccharomycotina* subphylum (Section 2.3) and synthesized de novo. All steps in the construction of integrative plasmids plasmid construction are shown in detail in Figure 1. DNA manipulations and restriction cloning were performed as in Sambrook (2001). Restriction and modification enzymes were used according to the manufacturer’s instructions (New England Biolabs, Ipswich, MA, USA). Competent cells of *E. coli* (NEB Stable, New England Biolabs, Ipswich, MA, USA) were prepared and electroporated as in Miller and Nickoloff (1995). Primers were synthesized by Metabion (Planegg/Steinkirchen, Germany). PCR was performed with Q5 polymerase (New England Biolabs, Ipswich, MA, USA), according to the manufacturer’s instructions.

#### 2.4.2. Construction of Replicative Plasmids

**Figure 2:**
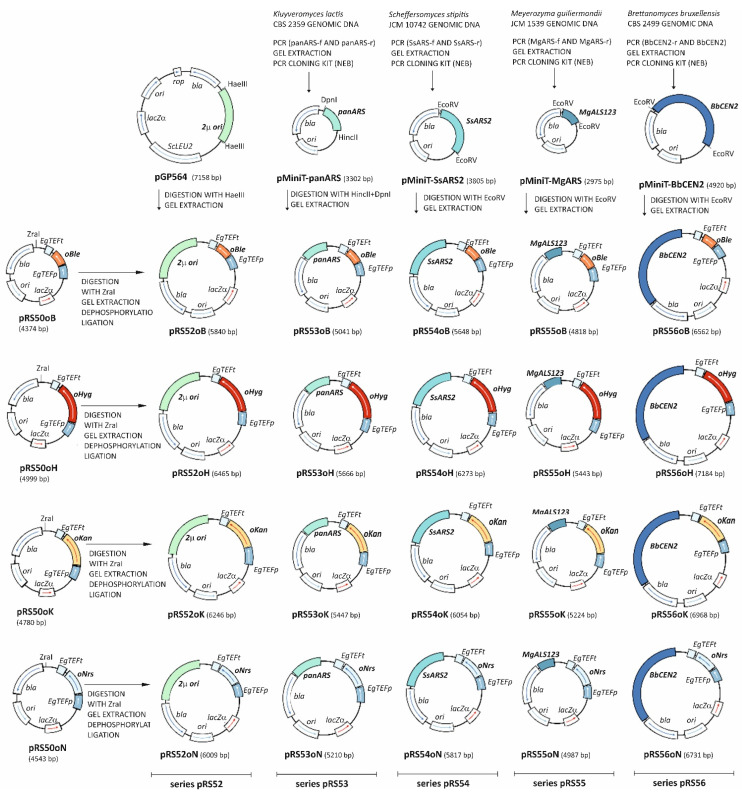
Construction of the plasmid series pRS52, pRS53, pRS54, pRS55, and pRS56 (replicative plasmids, each carrying one yeast replication origin) with a detailed list of intermediate cloning steps and relevant restriction sites. 2µ ori, panARS, SsARS2, MgALS123, BbCEN2 = yeast replication origins, ScLEU2 = LEU2 gene from *Saccharomyces cerevisiae*, rop = gene encoding Rop protein in *E. coli*. Other labels are identical to those in Figure 1.

To construct a set of replicative plasmids, the following origins of replication were used: *Saccharomyces cerevisiae* 2μ plasmid origin [15], *Kluyveromyces lactis* panARS origin [16], *Scheffersomyces stipitis Ss*ARS2 origin [17], *Merozyma guilliermondii Mg*ALS123 origin [18] and *Brettanomyces bruxellensis Bb*CEN2 origin [19]. Each of these origins was cloned in the backbone of all four yeast integrative plasmids, thus creating a set of 20 yeast replicative plasmids (series pRS52–pRS56). All steps in construction of replicative plasmids are shown in Figure 2.

### 2.5. Determination of Yeast Antibiotic Resistance

Liquid yeast culture was grown until the early stationary phase when 4 × 10^8^ cells were plated on the solid media supplemented with different concentrations of G418, hygromycin B, or clonNAT. As the yeasts were, in general. less sensitive to phleomycin, to determine their resistance to it, 4 × 10^7^ cells were plated on the solid media supplemented with different concentrations of phleomycin. Inoculated plates were incubated at 28 °C and checked for the spontaneously arising antibiotic-resistant colonies for up to 14 days, with plates containing less than five colonies after 14 days deemed as supplemented with antibiotic concentrations appropriate for the selection of the transformants. Minimal antibiotic concentrations, which suppress the growth of non-transformed yeasts on solid YPD medium and were used for selection of transformants is this work, are given in Table 2. *S. stipitis* and *M. guilliermondii* and easts belonging to the *Schwanniomyces* genus geneticin G418 did not suppress the growth of colonies even when the media contained 800 μg/mL of G418, which makes the geneticin resistance marker unsuitable for use in these yeasts.

### 2.6. Yeast Transformation and Molecular Analysis by Southern Blotting

The transformation of all yeast species in this work was performed using electroporation protocol described in [3], which was originally developed for transformation of *B. bruxellensis.* Yeast genomic DNA was isolated as in [20], and Southern blot was performed as in [21]. The digoxigenin-labelled probe was synthesized with the DIG DNA Labelling Mix (Roche, Mannheim, Germany), using as a template the PvuII-EcoRI fragment of pRS500 plasmid (Figure 1), thus labelling the gene bla and ori. The probe hybridizing with the DNA standard was synthesized using as a template the DNA of phage λ digested with HindIII.

## 3. Results and Discussion

### 3.1. Plasmid Set for Transformation of Yeasts Belonging to the Saccharomycotina Subphylum

The first aim of our work was to construct a set of integrative and replicative plasmids which could facilitate fundamental research as well as genetic transformation and strain construction for potential biotechnological applications for a broader range of various yeast species belonging to the *Saccharomycotina* subphylum.

**Figure 3:**
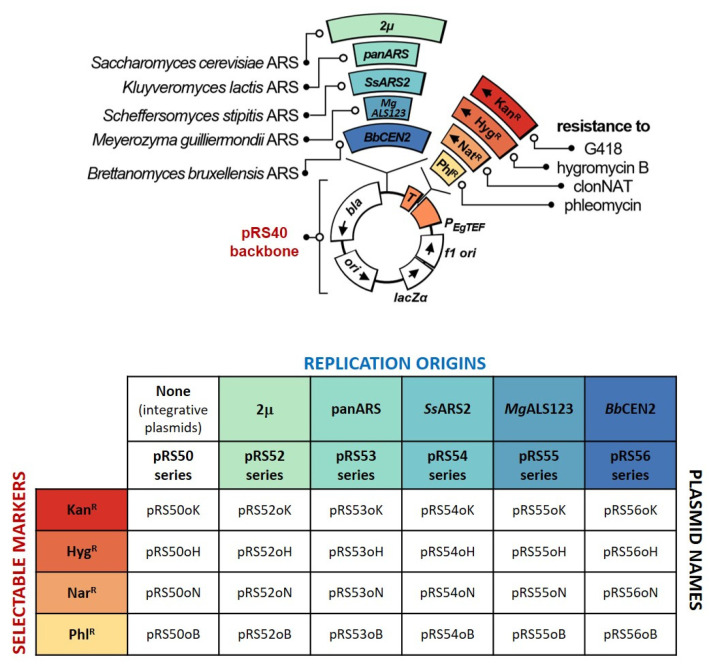
Schematic representation of integrative and replicative yeast plasmids constructed in this work and their names and main features. The pRS40 backbone carries sequences for selection and maintenance in *E. coli* (ori, bla) and other common vector features (*LacZα*, *f1 ori*), as well as TEF promoter (P*_Eg_*_TEF_) and terminator (T) from *Eremothecium gossypiii* for expression of antibiotic selectable markers in yeasts. Codon usage optimized markers conferring resistances to geneticin G418 (Kan^R^), hygromycin B (Hyg^R^), cloNAT (Nar^R^) or phleomycin (Phl^R^) were cloned under the regulation of the TEF promoter thus creating four different yeast integrative plasmids. Additionally, in each of these plasmids, the following origins of the replications were cloned: 2μ form *Saccharomycer cerevisiae*, panARS form *Kluyveromyces lactis*, *Ss*ARS2 from *Scheffersomyces stipitis*, *Mg*ALS123 form *Merozyma guilliermondii* or *Bb*CEN2 from *Brettanomyces bruxellensis*, thus creating a set of 20 replicative yeast plasmids.

The construction of all plasmids was based on pRS40B integrative yeast vector [13], which was used as a backbone (Figure 3). The pRS plasmid series is the most commonly used yeast shuttle vector, originally designed for *S. cerevisiae* and with several convenient features, including small size, high copy number replication origin in *E. coli*, a poly-linker with a plethora of unique restriction sites, LacZ for blue-white selection, and Amp^R^ *E. coli* selection and f1 origin [22,23]. Initially, the pRS series contained *HIS3*, *LEU2*, *URA3* and *TRP1* auxotrophic markers, but due to its popularity many variations and additional markers, both yeast and bacterial, have been combined with the pRS, as reviewed in [23]. The pRS40B plasmid which was used as a starting point for plasmid construction in this work was designed by Chee and Haase [13] and, among other features, contains a backbone with unique restriction sites for convenient expansion of the pRS plasmid series. All constructed plasmids, their names and important features are shown in Figure 3.

#### 3.1.1. Integrative Yeast Plasmids

To facilitate transformation of various yeast species across the *Saccharomycotina* subphylum, which is likely to exhibit different tolerances towards different antibiotics, four integrative plasmids were constructed (named pRS50 series; Figure 3), each carrying one of four common antibiotic resistance markers used in yeast (geneticin G415, hygromycin B, nourseothricin, also known as clonNAT, and phleomycin). Steps in construction of the integrative plasmids is described in detail in Section 2.4.1. To be functional across the *Saccharomycotina* subphylum the antibiotic markers were codon-optimized and synthesized de novo. Namely, alongside species which use the convectional genetic code, the *Saccharomycotina* subphylum also includes yeasts belonging to the CTG clade, for example most species within the biotechnologically relevant *Candida* genus. In the genetic code of these yeasts, the CTG encodes serine instead of leucine [24]. Hence, all existing in-frame CTG triplets were replaced with synonymous ones encoding leucine. Moreover, the overall codon usage of antibiotic markers was optimized to match that of *Candida albicans*, which will ensure sufficient expression in the yeasts within the CTG clade [25]. In the design of codon-optimized markers, the introduction of restriction sites was avoided and other modifications were introduced with the intent to increase the level of expression. The arginine encoding triplet CGA which can cause premature translation termination in *S. cerevisiae* [26] was replaced with synonymous codons. The AAAA sequence was also inserted directly before the AUG start codon, as this was shown to increase the level of expression in *S. cerevisiae* [27]. Clearly, some of the introduced modifications are based on evidence for increased level expression in a single yeast species. Such modifications might also have a positive effect on level of expression in some other yeast species of interest, but this cannot be claimed in advance without experimental evidence. DNA sequences of modified codon optimized antibiotic resistance markers which were used in this work are given in Supplementary Figure S1. Each of the constructed integrative plasmids successfully conferred antibiotic resistance when tested in *E. coli*, thus demonstrating that redesigned makers retained their functionality. It is important to note that, for each new yeast species, the sensitivity to available antibiotics will have to be experimentally tested and then a plasmid carrying an appropriate marker can be chosen for transformation.

#### 3.1.2. Replicative Yeast Plasmids

Replicative plasmids constructed in this work (series pRS52–pRS56, Figure 1) contain origins of five prominent yeast species from the *Saccharomycotina* subphylum—*Saccharomyces cerevisiae* 2μ plasmid origin [15], *Kluyveromyces lactis* panARS origin [16], *Scheffersomyces stipitis Ss*ARS2 origin [17], *Merozyma guilliermondii Mg*ALS123 origin [18], and *Brettanomyces bruxellensis Bb*CEN2 origin [19]. All of the chosen species are interesting from the biotechnological point of view, and additional tools which can facilitate research and strain constructions in these yeasts are valuable. Additionally, it has been shown that some of the chosen replication origins are active in various other species across the *Saccharomycotina* subphylum. For example, 2μ replication origin from *S. cerevisiae* is proven to be functional in *Torulospora delbrueckii*, *Torulospora pretoriensis*, *Sachwanniomycrs occidentalis*, *Pichia angusta*, *Pacchysolen tannophilus*, *Schizosaccharomyces pombe*, *Phaffia rhodozyma* [28], and *Eremothecium gossypii* [29], while panARS from *K. lactis* is shown to be functional in *Saccharomyces paradoxus*, *Saccharomyces bayanus*, *Naumovozyma castellii*, *Lachancea waltii*, *Lachancea kluyveri*, *Kluyveromyces wickerhamii*, *Ogatea polymorpha*, *Pichia pastoris* [16], and *Candida albicans* [30]. Although the other three origins of replications used in this work (*Ss*ARS2, *Mg*ALS123, *Bb*CEN2) are so far not known to be active in yeast species other than that of their origin, they have been much less extensively researched.

To test that cloned origins of replication retained their functionality, each of the five yeast species was transformed with corresponding plasmids, carrying its own replication origin. Prior to transformation, the appropriate antibiotic concentrations needed for selection of transformants to geneticin G418, hygromycin B, clonNAT and phleomycin were determined (Table 2). The transformation of all yeasts was performed identically, using the electroporation protocol previously described in [3] which was originally developed for transformation of *Brettanomyces bruxellensis*. However, we demonstrate here that it can be used in various species across the *Saccharomycotina* subphylum. Although it is impossible to say that it is universally applicable and might need an optimization for specific yeast species, this protocol seems the best available starting point when approaching transformation of a new and yet non-transformed budding yeast species. In general, although the chemical transformation method is commonly applied in *S. cerevisiae* as well as some other yeasts, we found that electroporation is commonly more efficient and more easily optimized. For example, the focus of some of our earlier studies was genetic transformation of notorious wine spoilage yeast *Brettanomyces bruxellensis*. Initially, we managed to achieve the efficiency of maximum 16 transformants/μg using the chemical method and 20 transformants/μg using electroporation [31]. However, optimization of the electroporation procedure resulted in an increase of transformation efficiency up to 2.8 × 10^3^ transformants/μg [3], while our attempts at optimization of chemical method were unsuccessful. Moreover, the same protocol we developed for *B. bruxellensis* resulted in a 10-fold increase of transformation efficiency of typical electroporation experiment of *S. cerevisiae.*

Additionally, a Southern blot hybridization of total undigested DNA isolated from yeast transformants was performed using DIG-labelled section of plasmid backbone (carrying *ori* and *bla*) as a probe. This analysis confirmed that in all cases plasmids are maintained in a circular form in yeast cells (Supplementary Figure S2). However, the Southern blot revealed a larger than expected plasmid present in some *B. bruxellensis* transformants as well as additional thin bands when any of the plasmids carrying the corresponding *Bb*CEN2 replication origin were isolated from *E. coli* (i.e., all plasmids within the pRS56 series), which was used as a control sample during Southern blotting. Upon further investigation, we determined that a somewhat different variant of *Bb*CEN2 origin was cloned into pRS56 plasmid series in this work than was previously reported in the literature [19]. The variant of *Bb*CEN2 replication origin cloned in this work contained two fairly large inverted repeats, each 410 bp long, separated by a 1350 bp long spacer region. We presume that the repeated DNA generated genetic instability and recombination in a certain number of plasmid molecules in vivo, giving rise to unexpected bands visible on Southern blotting. Nonetheless, it is clear that the cloned variant of *Bb*CEN2 origin is functional and plasmids carrying this origin are maintained in a circular form *in B. bruxellensis*.

### 3.2. Transformation of Yeasts Belonging to the Schwanniomyces Genus

The second aim of this work was to genetically transform some of the yeast species belonging to the *Schwanniomyces* genus (*S. pseudopolymorphus*, *S. polymorphus* var. *polymorphus* and *S. polymorphus* var. *africanus*). Prior to attempting transformation, sensitivity of the three selected species from the *Schwanniomyces* genus to antibiotics geneticin G418, hygromycin B and cloNAT was tested (Table 2). All three yeasts could tolerate high concentrations of G418, but both hygromycin B and cloNAT could be used for selection of transformants. Hence, in this work we chose to use plasmids carrying hygromycin B resistance marker (Hyg^R^), i.e., integrative plasmid pRS50oH and replicative plasmids pRS52oH (2μ origin), pRS53oH (panARS origin), pRS54oH (*Ss*ARS2 origin), pRS55oH (*Mg*ALS123 origin) and pRS56oH (*Bb*CEN2 origin). Prior to transformation the integrative plasmid was linearized with SacII restriction endonuclease which cuts the plasmid within the poly-linker sequence. Transformation efficiencies obtained in a typical experiment are shown in Table 3.

**Figure 4:**
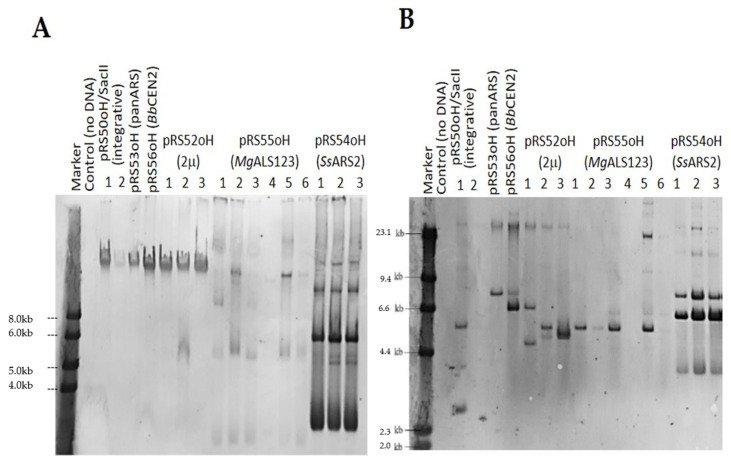
Typical results of molecular analysis by Southern blotting of undigested (**A**) and digested (**B**) DNA isolated form transformants of *S. polymorphus var. africanus*.

Transformants were obtained in all samples, with all types of transforming DNA, except *S. polymorphus* var. *africanus* transformed with pRS56oH (*Bb*CEN2 origin from *B. bruxellensis*). However, no colonies were observed on plates where control samples without DNA were plated, indicating that these were indeed true yeast transformants. To confirm this, total genomic DNA was isolated from several random colonies of each species obtained by transformation with each DNA, and molecular characterization by Southern blotting was performed. As a probe for hybridization, DIG-labelled plasmid backbone (containing *bla*, *ori and f1* regions) was used. For each sample, molecular analysis by Southern blotting was performed on undigested DNA (Figure 4A), as well as digested (Figure 4B) with XmnI (samples obtained by transformation with linearized integrative plasmid) or HindIII (samples obtained by transformation with replicative plasmids). Such digestion will either produce two bands of random sizes, if the transforming DNA integrated illegitimately into the genome, or one band of the same size as the plasmid used for transformation. in case the plasmid is maintained in circular form. Membranes shown on the figure below were obtained in the analysis of *S. polymorphus var. africanus* transformants, and similar results were obtained in the analysis of other two *Schwanniomyces* yeasts.

Molecular analysis by Southern blotting confirmed that colonies on all plates, obtained by all types of transforming DNA, were indeed true transformants, since they hybridized with DIG-labelled plasmid backbone. From the analysis of undigested DNA, it is clear that in case of linearized integrative plasmid, but also plasmids containing panARS, *Bb*CEN2 and 2μ replication origins transforming DNA integrated in the genome, while analysis of digested genomic DNA of same transformants demonstrates that a single plasmid molecule integrated at a random site in the genome. Additionally, for *S. pseudopolymorphus,* plasmid containing *Mg*ALS123 origin also integrated into the genome in a single copy. Single-copy integrations indicate that codon usage optimized hyg^R^ selectable marker is efficiently expressed and functional in yeasts belonging to the *Schwanniomyces* genus, since single copy of this marker is sufficient to confer normal growth of yeasts on medium containing hygromycin B. For all three yeast species, the efficiency of transformation (Table 3) was at least three times higher cases when linearized integrative DNA was used when compared to transformation with circular plasmids containing either panARS, *Bb*CEN2, or 2μ replication origins (as well as MgALS123 in case of *S. pseudopolymorphus*), which clearly cannot serve as a functional plasmid replication origin in these yeasts. However, surprisingly high numbers of transformants featuring essentially non-replicative circular plasmid integration into the genome could indicate that linear DNA might be relatively efficiently degraded in *Schwanniomyces*, or that the dynamics of the genome allows for efficient integration of circular non-replicative molecules, all coupled with a highly efficient transformation procedure which allows entry of DNA into a large number of competent cells.

Perhaps most interestingly, in all three yeasts molecular analysis demonstrated that plasmid containing *Ss*ARS2 replication origin is maintained in circular form. This is in accordance with the fact that in these cases very high efficiencies of transformation were achieved (up to 4.7 × 10^5^ transformants/μg; Table 4) which is comparable to efficiencies typically achieved when transforming model yeast *Saccharomyces cerevisiae* with replicative plasmid carrying its own origin (2μ or ARS). Moreover, the molecular analysis shows that in *S. polymorphus var. polymorphus* and *S. polymorphus var. africanus* plasmid carrying *Mg*ALS123 origin is also maintained in a circular form, which is also supported by high efficiency achieved during transformation with this plasmid (Table 3). However, colonies of transformants carrying plasmid pRS55oH (*Mg*ALS123) were relatively small and slow growing, while bands visible on Southern blot membranes are relatively pale, all indicating that this plasmid might not be as stable as pRS54oH (*Ss*ARS2) and, possibly, is frequently lost during culture growth.

To experimentally test the stability of plasmids which are maintained in circular form in the genomes of *Schwanniomyces* yeasts, individual transformants were inoculated in liquid medium supplemented with hygromycin B and grown for 48 h until the stationary phase. Cultures were then plated on complete medium with and without hygromycin B to determine the percentage of plasmid-containing cells. The results are shown in Table 4. As a control, the percentage of hygromicin B resistant colonies after cultivation under selective pressure was determined for transformants carrying pRS50oH plasmid integrated at a random site in the genome.

As expected, when integrated into the genome, plasmids were completely stably inherited during culture growth. The stability of plasmid pRS54oH carrying *Ss*ARS2 origin greatly varied between individual *Schwanniomyces* species. In *S. polymorphus var. africanus* this plasmid was completely stable, indicating that it might be present in a larger number of copies per individual cell. This idea is supported by results of molecular analysis by Southern blotting (Figure 2), which showed dark and thick bands corresponding to pRS54oH (SsARS2) in transformants of *S. polymorphus var. africanus.* Taken together, stability and most likely larger plasmid copy-number per cell make pRS54oH (SsARS2) an ideal replicative vector for use in *S. polymorphus var. africanus*. In *S. polymorphus var. polymorphus*, the plasmid is also relatively stable, but much less so in *S. pseudopolymorphus*. The plasmid pRS55oH carrying *Mg*ALS123 replication origin is far less stable than pRS54oH (*Ss*ARS2) and thus less suitable to use as a replicative plasmid in *S. polymorphus var. polymorphus* and *S. polymorphus var. africanus*.

It is perhaps not surprising that, of all five tested replication origins, the *Ss*ARS2 which originates from *Scheffersomyces stipits* is that which retains most of its functionality in yeasts belonging to the *Schwanniomyces* genus, since *Scheffersomyces* and *Schwanniomyces* genera are the most closely related [5]. Hopefully, the five replication origins which have been cloned on a plasmid set in this work coupled with several codon optimized antibiotic selectable marker will open the possibility for transformation of a larger number of yet unresearched species in the *Saccharomycotina* subphylum. Moreover, we demonstrated here that the electroporation protocol developed earlier for *B. bruxellensis* (Miklenić et al., 2015) can be applied to a number of budding yeast species (*S. cerevisiae*, *S. stipitis*, *M. guilliermondi*, *K. lactis*), including the three selected species from the *Schwanniomyces* genus, where very high transformation efficiencies were achieved without any optimization or modification of the original protocol.

## Figures and Tables

**Table 1:** Antibiotic concentrations which can be used for selection of yeast transformants on solid YPD media.

	Geneticin G418 [μg/mL]	Hygromycin B [μg/mL]	cloNAT [μg/mL]	Phleomycin [μg/mL]
*S. cerevisiae*	200	300	100	10
*K. lactis*	150	200	20	100
*S. stipitis*	selection not possible	600	50	100
*M. guilliermondii*	selection not possible	500	200	200
*B. bruxellensis*	250	150	35	150
*S. pseudopolymorpshus*	selection not possible	200	50	Not determined
*S. polymorphus var. polymorphus*	selection not possible	200	50	Not determined
*S. polymorphus var. africanus*	selection not possible	400	50	Not determined

**Table 2:** List of yeast strains transformed in this study and of plasmids and oligonucleotides used as material.

Yeast Strains	Genotype	Reference
*Saccharomyces cerevisiae* BY 4742	MATα his3Δ1 leu2Δ0 lys2Δ0 ura3Δ0	[11]
*Kluyveromyces lactis* CBS 2359T	Type strain	Westerdijk Fungal Biodiversity Institute, The Netherlands
*Scheffersomyces stipitis* JCM 10742T	Type strain	Japan Collection of Microorganisms, Japan
*Meyerozyma guilliermondii* JCM 1539T	Type strain	Japan Collection of Microorganisms, Japan
*Brettanomyces bruxellensis* CBS 2499		Westerdijk Fungal Biodiversity Institute, The Netherlands
*Schwanniomyces pseudopolymorphus* JCM3652^T^	Type strain	Japan Collection of Microorganisms, Japan
*Schwanniomyces polymorphus var. polymorphus* JCM3647^T^	Type strain	Japan Collection of Microorganisms, Japan
*Schwanniomyces polymorphus var. africanus* JCM7443^T^	Type strain	Japan Collection of Microorganisms, Japan
**Plasmids**	**Feature Important for This Study**	**Reference**
pGP564	contains 2µ replication origin	[12]
pRS40B *	source of the pRS40 backbone	[13]
pRS50 series (pRS50oK, pRS50oH, pRS50oN, pRS50oB)	Integrative plasmids with codon-optimized selectable markers (Kan^R^, Hyg^R^, Nar^R^, Phl^R^)	This study
pRS52 series (pRS52oK, pRS52oH, pRS52oN, pRS52oB)	Contains 2μ replication origin from *S. cerevisiae* and codon-optimized selectable markers (Kan^R^, Hyg^R^, Nar^R^, Phl^R^)	This study
pRS53 series (pRS53oK, pRS53oH, pRS53oN, pRS53oB)	Contains panARS replication origin from *K. lactis* and codon-optimized selectable markers (Kan^R^, Hyg^R^, Nar^R^, Phl^R^)	This study
pRS54 series (pRS54oK, pRS54oH, pRS54oN, pRS54oB)	Contains SsARS replication origin from *S. stipitis* and codon-optimized selectable markers (Kan^R^, Hyg^R^, Nar^R^, Phl^R^)	This study
pRS55 series (pRS55oK, pRS55oH, pRS55oN, pRS55oB)	Contains MgALS123 replication origin from *M. guilliermondii* and codon-optimized selectable markers (Kan^R^, Hyg^R^, Nar^R^, Phl^R^)	This study
pRS56 series (pRS56oK, pRS56oH, pRS56oN, pRS56oB)	Contains BbCEN2 replication origin from *B. bruxellensis* and codon-optimized selectable markers (Kan^R^, Hyg^R^, Nar^R^, Phl^R^)	This study
**Oligonucleotides**	**Sequence**	**Reference**
panARS-f	gtgaggtaccgaaggaatttgctgttatggag	This study
panARS-r	gtgaggtaccactgacactgttgactctg	This study
SsARS2-f	gatatccagaataattgatggtccgc	This study
SsARS2-r	gatatctggattgttgtgctctcg	This study
MgARS-f	gatatcagatgacaagcccaaacac	This study
MgARS-r	gatatccatatgtccttgccagttgaacca	This study
DbCEN2-f	gatatcctgaggttgctaagcccc	This study
DbCEN2-r	gatatcgtgaatagtgaagccaactggt	This study
AgTEF-f	aggcctcccgggacatggaggcccagaat	This study
AgTEF-r	aggcctcccgggcagtatagcgaccagcattc	This study

* pRS40B was a gift from Steven Haase (Addgene plasmid # 35478; http://n2t.net/addgene:35478 (accessed on 24 April 2022); RRID:Addgene_35478).

**Table 3:** Transformation efficiencies obtained during a typical transformation of yeasts from the *Schwanniomyces* genus with linearized integrative plasmid and replicative plasmids carrying various replication origins. Control samples where no DNA was added in the transformation mixture did not yield any colonies.

Transformation Efficiency (Transformants/μg)						
	pRS50oH/SacII (Linear)	pRS52oH (2μ Origin)	pRS53oH (panARS Origin)	pRS54oH (*Ss*ARS2 Origin)	pRS55oH (*Mg*ALS123 Origin)	pRS56oH (*Bb*CEN2 Origin)
*S. pseudopolymorphus*	8.1 × 10^3^	424	21	4.7 × 10^5^	32	1.1 × 10^3^
*S. polymorphus var. polymorphus*	952	360	117	3.7 × 10^5^	More than 2 × 10^4^	714
*S. polymorphus var. africanus*	476	88	149	2.7 × 10^5^	More than 2 × 10^4^	0

**Table 4:** Stability of replicative plasmids pRS45oH (*Ss*ARS2), pRS55oH (*Mg*ALS123) and pRS50oH plasmid integrated into the genome (control) in yeasts belonging to the *Schwanniomyces* genus. The plasmid stability is determined as a percentage of cells resistant to hygromicin B after cultivation till stationary phase under hygromicin selective pressure.

Plasmid Stability			
	pRS50oH Integrated in the Genome	pRS54oH (*Ss*ARS2 Origin)	pRS55oH (*Mg*ALS123 Origin)
*S. pseudopolymorphus*	100%	5.9%	Not replicative
*S. polymorphus var. polymorphus*	100%	28%	1%
*S. polymorphus var. africanus*	100%	100%	0.2%

## Data Availability

Not applicable.

## Supplementary Materials

The following supporting information can be downloaded at: https://www.mdpi.com/article/10.3390/jof8050531/s1, Supplementary Figure S1: Sequences of de novo synthesised ORFs encoding antibiotic resistance markers. Supplementary Figure S2: Genetic analysis of yeast transformants by Southern blot of the undigested genomic DNA, with digoxigenin-labelled probe hybridising to genes bla and ori present in the plasmid backbones.
