# Supplementary material for: Toolbox for Genetic Transformation of Non-Conventional Saccharomycotina Yeasts: High Efficiency Transformation of Yeasts Belonging to the Schwanniomyces Genus
Source: J Fungi (Basel). 2022 May 20;8(5):531. doi: 10.3390/jof8050531 (PMC9146037; doi:10.3390/jof8050531)
Supplement: Supplementary file 1 [file jof-08-00531-s001.zip › jof-1719293-supplementary.pdf]

Supplementary figures

> oBle
atgggaatgacagatcaggccacccctaatcttccaagtagagactttgatccaactgcagccttttacgagcgtttggggtttggtatagtgttccgtgacgctgggtggatg
atacttcagcgtggggatttaatgcttgagttctttgctcatcctggacttgacccattagcttcctggttctcctgttgtttgcgtcttgacgatttggccgaattttacagacaatgt
aagagtgtcggaatacaggaaacttcctctggttatcctagaatacacgctcctgagttgcaagagtgggggggaacaatggctgctttagtagatcctgacggaactttatt
acgtcttatccagaatgaacttcttgcaggaatatcttga
> oHyg
atgggaaagaaacctgaacttactgcaacatccgtcgaaaagtttttaattgagaaatttgatagtgtgtccgaccttatgcaacttagtgaaggagaagagtcaagagctttt
agttttgatgtaggaggacgtggttatgtcttgagagtcaatagttgtgcagatggattctacaaggatcgttatgtctatcgtcatttcgcctccgccgccttaccaatccctgaa
gtcttagacataggggagttctcagagtccttaacttactgcatatcccgtagagcacaaggagttactcttcaggacttgccagagacagaattacctgccgtccttcagccag
ttgccgaggcaatggacgctatagccgctgcagacttatcccaaactagtggattcgggccatttggtcctcaaggaattgggcaatatactacatggcgtgatttcatctgcg
caatagcagaccctcatgtctaccattggcagaccgtaatggacgatactgtatccgcttctgtggcacaagcacttgacgagttaatgttgtgggcagaggattgtccagaa
gtcagacatttggtgcacgctgattttggtagtaacaatgtgcttaccgacaatggtcgtataaccgcagtcatcgattggtcagaagctatgttcggggattcacagtacgag
gtagccaatatttttttctggagaccttggcttgcatgtatggaacagcagacccgttacttcgaacgtagacacccagaattagccgggtctccacgtttaagagcttatatgtt
gagaattggattagaccaactttatcaatctttagtcgatggaaatttcgacgatgccgcttgggcccaaggtagatgcgacgccattgtcagaagtggagctggaactgtgg
ggcgtacccaaattgcccgtagatcagccgcagtgtggacagacggatgcgtggaagtattagcagatagtgggaatcgtcgtccttccacacgtccaagagccaaggaat
aa
> oKan
atgggtaaggagaagacacacgtgagtcgtccaagattgaacagtaacatggacgctgatttatacgggtataaatgggctcgtgataatgttggtcagtcaggagccaca
atctatagattgtacggaaagcctgacgccccagaattgtttttgaagcatggtaagggttccgtcgccaatgatgttacagacgaaatggtgagacttaattggttgaccgag
ttcatgcctttacctaccatcaaacacttcataagaacacctgacgacgcatggttacttacaacagctatccctggtaagactgcattccaggttcttgaggagtatccagactca
ggtgagaacattgtcgatgccttagcagtgtttcttcgtagattacattcaataccagtttgtaattgccctttcaattctgatagagtgtttcgtcttgctcaagctcagtctcgtatg
aataatggtttggtggatgcttccgacttcgacgacgaacgtaacgggtggccagttgaacaagtctggaaagaaatgcacaaattattaccattctcacctgattcagtggtg
actcacggtgatttctcacttgacaatttgatatttgatgaaggtaaattaatagggtgcatagatgttgggagagtcggtatagccgaccgttaccaagatttagctattctttg
gaattgtttgggggagttcagtccttcacttcagaaacgtttatttcagaagtacggaatagacaatccagacatgaacaagttgcagtttcacttaatgttggacgaatttttcta
a
> oNrs
atggggaccacattagatgacaccgcatatcgttaccgtacatccgtcccaggagacgccgaagctattgaggcacttgacggatcattcacaaccgacacagtatttagagt
taccgctacaggtgatggattcacccttcgtgaggtgccagtagatccaccacttaccaaagtatttccagatgacgagagtgacgatgagagtgacgatggggaggacgg
agacccagactccagaacctttgtcgcctatggagacgatggtgatttggctggtttcgtcgttgtctcttactcaggatggaacagacgtttaactgtggaggacatcgaagtt
gctccagagcacagaggacatggggtaggacgtgctttaatgggattagctactgagttcgcccgtgagcgtggagctggtcatttgtggttggaagtgactaacgtgaac
gcacctgccatacatgcataccgtagaatgggattcactctttgtggtttagataccgcattgtatgacggaaccgcatctgatggtgagcaagccttatatatgtctatgccatg
cccttaa

Supplementary Figure 1. Sequences of *de novo* synthesised ORFs encoding antibiotic resistance markers.

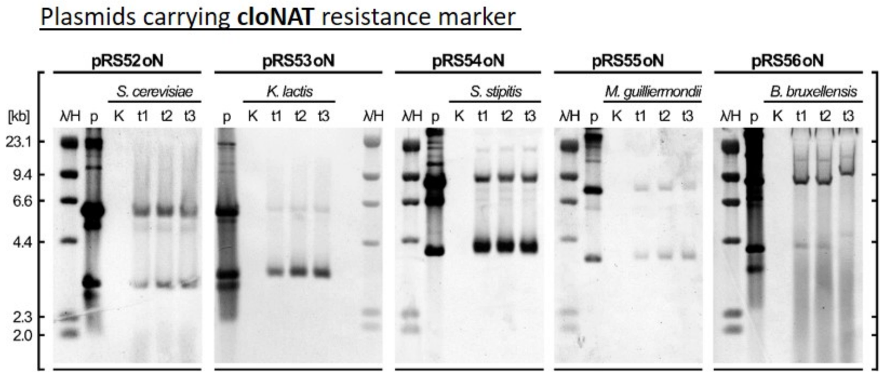

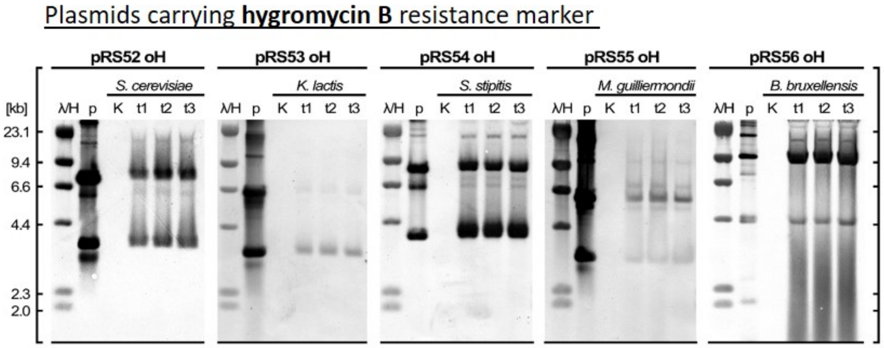

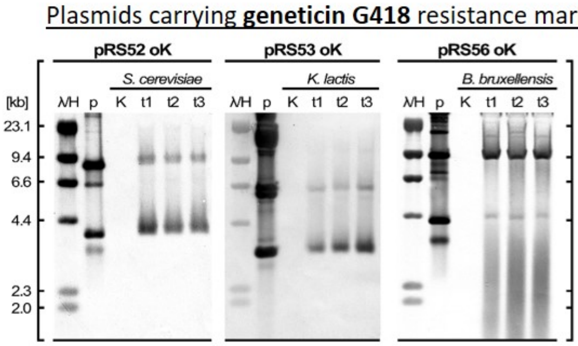

* Selection is of *S. stipitis* and *M. guilliermondii* transformants is not possible on geneticin G418

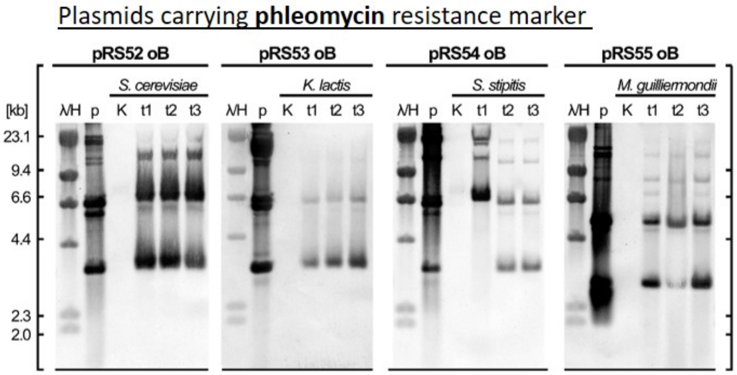

* Transformants of *B. bruxellensis* were not obteined when selection on pleomycin was used

Supplementary Figure 2. Genetic analysis of yeast transformants by Southern blot of the undigested genomic DNA, with digoxigenin-labelled probe hybridising to genes bla and ori present in the plasmid backbones. Each of the membranes focuses on the combination of one yeast species (columns) and its replicative plasmids conferring resistance to cloNAT, hygromycin B, G418, or phleomycin (rows). λ/H = marker (phage λ DNA digested with HindIII), p = replicative plasmid isolated from E. coli, K = untransformed yeast strain, t1-t3 = three yeast transformants.
